# Relationship between self-esteem and quality of life in middle-aged and older patients with chronic diseases: mediating effects of death anxiety

**DOI:** 10.1186/s12888-023-05459-4

**Published:** 2024-01-02

**Authors:** Pengjuan Ji, Lin Zhang, Ziyun Gao, Qiqi Ji, Jiashuang Xu, Yian Chen, Miaojing Song, Leilei Guo

**Affiliations:** 1 School of Nursing, Jinzhou Medical University, No.40, Section 3, Songpo Road, Linghe District, Jinzhou City, Liaoning Province P.R. China; 2 Department of Internal Medicine Nursing, School of Nursing, Wannan Medical College, 22 Wenchang West Road, Higher Education Park, Wuhu City, An Hui Province P.R. China

**Keywords:** Middle-aged and older adults, Chronic diseases, Self-esteem, Quality of life, Death anxiety

## Abstract

**Background:**

Many studies have explored the relationship between self-esteem and quality of life. However, few studies have elucidated the mechanisms underlying the relationship between self-esteem and quality of life in middle-aged and older patients with chronic diseases. The present study aimed to explore the mediating role of death anxiety in this relationship.

**Methods:**

Middle-old-aged patients with chronic diseases were selected as the respondents by using a multi-stage sampling method, random number table method from October 2021 to February 2022 in the Second Affiliated Hospital of Zhejiang University School of Medicine. The Cumulative Disease Rating Scale, the Self-Esteem Scale (SES), the Chinese version of the Death Anxiety Scale (CT-DAS), and the Simplified version of the Quality of Life Scale (SF-12) were used as the researching tools to conduct the survey. SPSS26.0 was used to analysis data. AMOS 23.0 software was used to construct structural equation modeling.

**Results:**

294 valid questionnaires were collected. There were significant differences in quality of life among middle-aged and elderly patients with chronic diseases who have different physical activities, socialization, and chronic pain (*P* < 0.01); Self-esteem was positively associated with quality of life (r = 0.330, *P* < 0.01), self-esteem was negatively associated with death anxiety (r = -0.222, *P* < 0.01), and death anxiety was negatively associated with quality of life (r = -0.263, *P* < 0.01); Death anxiety partially mediated the relationship between self-esteem and quality of life, with the mediating effect accounting for 18.40% of the total effect.

**Conclusion:**

Death anxiety partially mediates the relationship between self-esteem and quality of life. Interventions to improve self-esteem and reduce death anxiety should be used to improve the quality of life of middle-aged and senior patients with chronic diseases.

## Background

Around the world, chronic diseases are a major public health concern. In practically every nation, the prevalence of chronic diseases, including cancer, diabetes, and cardiovascular disease, is on the rise [1]. China currently has more than 260 million chronic illness patients, and as the audience gets younger due to lifestyle changes, it is predicted that the proportion of patients over 40 will increase by three to four times by 2030 [2]. Due to the lengthy duration and irreversible etiology of the disease, the restriction of everyday activities, and the limited somatic mobility, middle-aged and elderly patients with chronic diseases are more likely to experience negative feelings [3]. All of these have a negative impact on their quality of life.

Quality of life (QoL), which assesses the impact of health conditions on daily life based on an individual’s self-perception and sociocultural context, is now widely used as an important measure of health status [4]. Numerous variables, such as psychological, physical, social functioning, and interpersonal interactions, have an impact on one’s quality of life. Among psychological factors, self-esteem, as an internal coping mechanism, directly affects patients’ quality of life [5]. Self-esteem is viewed as a self-evaluation that develops during a person’s maturation process and is critical for upholding healthy relationships with aspects like a person’s emotions, behaviors, and so on [5]. People with chronic illnesses can gain several benefits from raising their levels of self-esteem. High self-esteem encourages adaptive persistent actions when unfavorable or unpleasant emotions pose harm to one’s mental health, and people are more likely to take protective measures to decrease the danger and maintain a high quality of life [6–8]. Based on the correlation between quality of life and self-esteem, Chia-Chun Li’s study showed that self-esteem was a significant independent predictor of overall quality of life [9]. Additionally, self-esteem in older people had a protective effect on quality of life, as demonstrated by a study by Edison Vitorio de Souza Junior [10]. Investigating the effect of self-esteem on quality of life in middle-aged individuals with chronic conditions is therefore important.

Due to the serious negative consequences of ongoing diseases and the unpredictable nature of death, middle-aged and elderly patients with chronic illnesses are also more vulnerable to death anxiety. Death anxiety is an emotional condition of anxiety and fear brought on by thoughts of dying [11, 12]. It affects our life experiences and behavioral patterns. People’s quality of life suffers when they become aware of the potentially detrimental behavioral and emotional effects of death anxiety. The Theory of Fear Management (TMT) claims that self-esteem is one of the coping strategies people use to deal with their fear of dying [13–15]. People with strong self-esteem will defend themselves against death anxiety by abiding by their cultural norms and values [13], protecting themselves from harm, and maintaining their quality of life when they directly think about death or experience death anxiety. On the contrary, if a person’s self-esteem is dangerously low, they might not be able to control the negative effects of death fear and might even speed them up, which would reduce their quality of life [16].

**Fig. 1:**
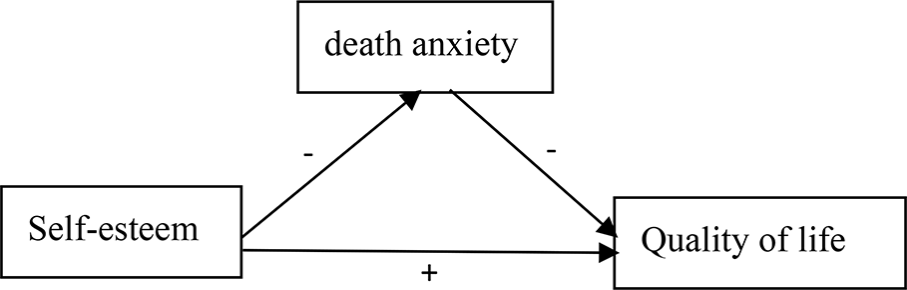
Hypothetical model

Quality of life and death anxiety have been linked in previous studies [17–19], as have self-esteem and death anxiety [16, 20, 21], as well as self-esteem and quality of life [9, 10, 22]. The three’s relationship has nevertheless received little discussion. Therefore, in the middle-aged and older chronic disease groups, we hypothesized that H1a: Self-esteem and quality of life are positively correlated; H1b: Self-esteem and death anxiety are negatively correlated; H1c: Death anxiety and quality of life are negatively correlated. H2: Death anxiety mediates the relationship between patients’ self-esteem and quality of life. The hypothetical model for this study is shown in Fig. 1. To investigate this association, we used regression analysis and structural equation modeling (SEM) to identify the mediating effect and construct the SEM hypothesis model. Aims to provide a theoretical basis for clinical healthcare professionals to improve the quality of life of middle-aged and elderly patients with chronic diseases.

## Methods

### Participants

Using multi-stage sampling and the random number table approach, this study gathered data in the Shang Cheng District of Hangzhou City, Zhejiang Province. First, a third-class hospital from the Shang Cheng District was randomly chosen: the Second Affiliated Hospital of Zhejiang University School of Medicine. Second, survey stations were set up in each of the three departments that were randomly picked. Finally, chronic patients were randomly chosen for the questionnaire survey. For sample size calculations, we employed an application developed by Schoemann in the statistical computing language R, which was based on the Monte Carlo confidence interval power analysis approach [23, 24]. The required parameters were entered sequentially and several runs were performed to ensure stability of the results. When the conventional power level of 0.80 was chosen, the minimum sample size required was 222. To reduce the probability of making mistakes, we increased the sample size to 320 (Power = 0.95). After eliminating missed and invalid questionnaires, we finally recovered 294 (Power = 0.92) valid questionnaires, with an effective recovery rate is 91.87%. The following criteria were used to determine who should be included: (i) Age 45 years, satisfied diagnostic criteria for chronic diseases; (ii) clear consciousness; (iii) informed of the diagnosis; and (iv) consented to participate. Exclusion criteria include: (i) patients with serious mental illnesses who are unable to communicate and express themselves normally; and (ii) patients who refuse to cooperate with this study.

Prior to the survey, investigators were given standardized training to make the scoring criteria and communication skills clear. The patient was given questionnaires to complete on their own after the researchers had obtained their informed consent. Researchers conducted in-person interviews and filled out questionnaires with dyslexics. The Declaration of Helsinki is followed by the application of all techniques.

### Research instruments

#### Demographics

Eight demographic questions were asked of the participants: age, gender (male or female), marital status (married, single/divorced/other), education level (primary school, middle school, high school or technical secondary school, junior college, bachelor degree or above), monthly income status, level of physical activity (regular/irregular/none), level of social activity (often/occasional/none), and chronic pain (yes or no).

#### Cumulative illness rating scale (CIRS -G)

The scale was created by William L.Leidy along with others in 1980 [25], and it was a pertinent rating scale that took into account every physiological system [26]. The severity of the condition is determined by a score of 0–4 (none–extremely severe), and there are 14 items in total. Each entry corresponds to the relevant organ system. Scores range from 0 to 56. It was solely employed in this study to assess the disease’s prevalence in older and middle-aged persons.

#### Self-esteem scale(SES)

The SES scale was compiled by Rosenberg in 1965 [27]. It was used to assess the level of self-esteem of middle-aged and elderly chronic patients, which has been widely used in China [28]. Ten questions make up the scale, which is graded on a 4-point scale. Top grade completely complies with the requirement, Class 2 complies with it, Class 3 indicates that it does not, and Class 4 indicates that it is very out of compliance. Five of the items indicate positive self-esteem, whereas five indicate negative self-esteem. The total score is on a scale from 0 to 40, with a greater score indicating a higher level of self-esteem. The scale’s Cronbach’s alpha in this study was 0.848.

#### Chinese templer -death anxiety scale (CT- DAS)

Templer created the Death Anxiety Scale in 1970 [29], and Yang Hong et al. cross-culturally altered it to create the Chinese version of the Death Anxiety Scale (CT-DAS) [30]. The scale has 4 dimensions and 15 items on a 5-point Likert scale from 1 to 5 (strongly disagree to strongly agree). Items 2, 3, 5, 6, 7 and 15 are reverse scored. The scale total is the sum of the scores for each item, with a score range of 15–75; a score of > 35 is considered to be an intense case of death anxiety [31]. The scale’s Cronbach’s alpha in this study was 0.812.

#### Quality of life scale (SF-12)

The Quality of Life Scale (SF-12) is an abridged version of SF-36 with good reliability and validity [32, 33]. The scale consists of 12 items, including 8 dimensions of General Health (GH), Physiological Function (PF), Role Physical (RP), Bodily Pain (BP), Role Emotional (RE), Mental Health (MH), Vitality (VT), and Social Function(SF), and the summation of scores on the four dimensions of GH, PF, RP, and BP constituted the Physical health dimension (PCS), while scores on the four dimensions of RE, MH, VT, and SF were summed to form the Mental health dimension (MCS). The higher the score, from 0 to 100 (from bad to truly exceptional), the higher the quality of life. The scale’s Cronbach’s alpha in this study is 0.863.

### Data analysis

Using the SPSS 26.0 program, a database was generated and statistically examined. The data was entered and confirmed by two researchers. On the basis of the participants’ overall characteristics, self-esteem, death anxiety, and quality of life, descriptive statistics were generated. Independent t-tests and one-way analyses of variance were used to examine differences in quality of life based on general characteristics. Correlations between quality of life, death anxiety, and self-esteem were examined using Pearson’s correlation coefficient and scatter plots. Regression analysis was used to predict the relationship between self-esteem and quality of life, as well as the relationship between self-esteem and quality of life when death anxiety was added. Using AMOS 23.0 software, path analyses between variables were performed, and structural equations were constructed. The model was considered appropriate when the fit indices TLI, and CFI > 0.9 and RMSEA and SRMR < 0.8 [34, 35]. And the mediating effect of death anxiety was verified using the Bootstrap program.

## Results

### Participants’ characteristics

Table 1 displayed the demographic information about the study population as well as the univariate analyses of the study participants’ quality of life under various conditions. Out of 294 patients with chronic diseases, the most common diseases were vascular diseases, endocrine/metabolic/breast cancer, liver disease, heart disease, and respiratory diseases. 108 (36.74%) were female and 186 (63.26%) were male; the age range of the participants was 45–96 years with a mean age of 64.06 ± 10.23 years; the majority of the patients were at secondary school level or below (68.02%); 57.82% of the patients reported irregular physical activity; and 30.27% of the participants expressed having chronic pain. Quality of life was statistically different in terms of physical activity, socialization, and chronic pain. Further details are shown in Table 1.

**Table 1:** Univariate analysis quality of life of chronic patients with different characteristics (*N* = 294)

Variables	Group	N (%)	Mean ± SD	*F/t*	*P*
Sex	Male	186 (63.2%)	75.57 ± 9.45	0.285	0.594
	Female	108 (36.7%)	74.92 ± 11.13		
Age	45~	118 (40.1%)	76.77 ± 9.771	2.104	0.124
	60~	134 (45.6%)	74.52 ± 10.43		
	75~	42 (14.3%)	73.85 ± 9.56		
Marital status	Married	284 (96.6%)	75.44 ± 10.07	0.616	0.541
	Single, Divorce, Other	10 (3.4%)	72.47 ± 10.88		
Educational Level	Primary school	110 (37.4%)	74.98 ± 9.81	1.066	0.374
	Middle school	90 (30.6%)	74.42 ± 10.17		
	High school or technical secondary school	50 (17.0%)	75.54 ± 9.96		
	Junior college	22 (7.5%)	76.53 ± 12.34		
	Bachelor degree or above	22 (7.5%)	79.10 ± 8.74		
Monthly income	< 1000	31 (10.5%)	73.64 ± 9.15	1.482	0.196
	1000~	64 (21.7%)	74.45 ± 11.33		
	3000~	99 (33.6%)	75.78 ± 10.29		
	5000~	57 (19.4%)	74.03 ± 9.50		
	7000~	24 (8.1%)	77.57 ± 8.03		
	10,000~	19 (6.4%)	79.76 ± 9.27		
Physical activity	Regular	82 (27.9%)	79.16 ± 9.49	14.502	< 0.001
	Irregular	170 (57.8%)	74.85 ± 9.63		
	None	42 (14.3%)	69.41 ± 10.09		
Social contact	Often	64 (21.8%)	79.37 ± 9.43	9.421	< 0.001
	Occasionally	173 (4.4%)	75.03 ± 9.92		
	None	57 (19.4%)	71.69 ± 9.86		
Chronic pain	Yes	89 (30.3%)	71.09 ± 9.03	24.287	< 0.001
	No	205 (69.7%)	77.17 ± 9.98		

### Statistical description and pearson correlation analysis of variables

**Fig. 2:**
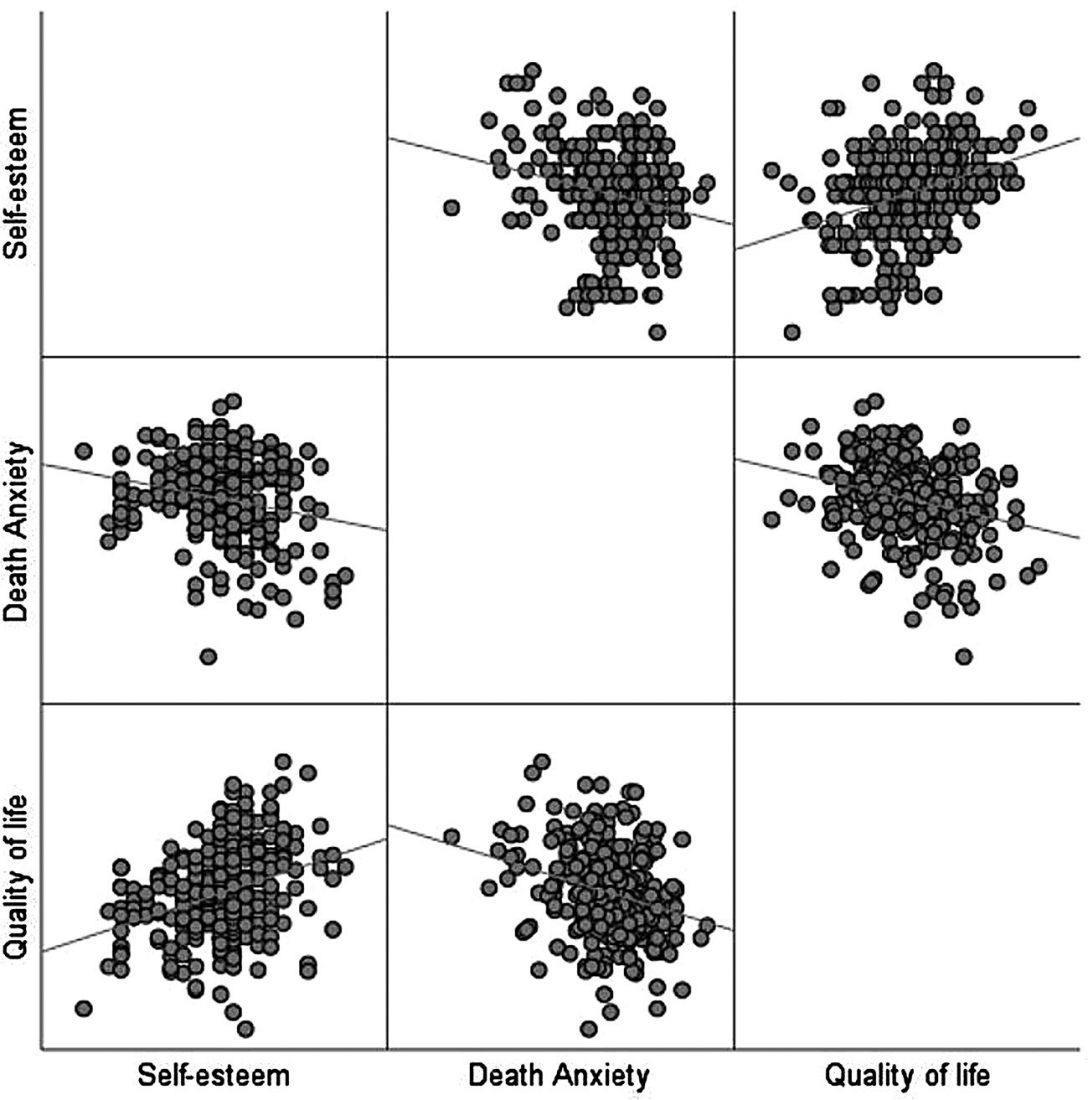
Scatterplot of relationship between variables

Middle-aged and elderly chronic patients’ PCS scores (36.96 ± 7.97) and MCS scores (38.37 ± 6.64) for quality of life were worse than the normative PCS scores (51.20 ± 6.60) and MCS scores (49.90 ± 7.70) [36]. The death anxiety score was high (46.91 ± 8.97 > 35). As shown by the scatter plot, there was a linear relationship between the variables. Further correlation analysis showed that self-esteem was positively correlated with quality of life and negatively correlated with death anxiety; death anxiety was negatively correlated with quality of life. Detailed information is shown in Fig. 2; Table 2.

**Table 2:** Descriptive statistics and correlation analysis of death anxiety, self-esteem and quality of life

	Mean ± SD	Quality of Life	Self-esteem	Death Anxiety
Quality of life	75.33 ± 10.08	1		
Self-esteem	28.13 ± 3.57	0.330**	1	
Death anxiety	46.91 ± 8.97	-0.263**	-0.222**	1

*Note*: **represents *P* < 0.01

### Analysis of multiple linear regression

Three multiple linear regression analyses were performed, with self-esteem and death anxiety serving as the primary independent variables, demographic characteristics serving as the control variables, and quality of life as the dependent variable. The results showed (Table 3) that according to model 1, general information explained 14.20% of the standardized variance (F = 7.041 *P* < 0.01); multiple-linear regression analysis in model 2 showed that self-esteem significantly and positively predicted quality of life, explaining 21.90% of the standardized variance (F = 10.152, *P* < 0.01); and model 3 added death anxiety to model 2, which negatively predicted quality of life and explained 25.60% of the standardized variance (F = 11.056, *P* < 0.01).

**Table 3:** Multiple linear regression analysis results

Variable		Model1				Model2				Model3			
		B	SE	t	95%CI	B	SE	t	95%CI	B	SE	t	95%CI
General Information	Age	-0.913	0.807	-1.131	-2.501, 0.676	-1.121	0.770	-1.455	-2.637, 0.395	-0.691	0.761	-0.909	-2.188, 0.806
	Gender	-0.135	1.157	-0.117	-2.412, 2.142	0.152	1.104	0.138	-2.021, 2.326	0.600	1.085	0.553	-1.536, 2.735
	Marital status	-1.483	1.590	-0.933	-4.613, 1.647	-1.924	1.519	-1.267	-4.913, 1.065	-1.179	1.496	-0.789	-4.123, 1.765
	Educational Level	-0.369	0.572	-0.646	-1.496, 0.757	-0.634	0.548	-1.157	-1.713, 0.444	-0.841	0.538	-1.563	-1.899, 0.218
	Monthly income	-0.281	0.528	-0.533	-1.321, 0.758	-0.403	0.504	-0.799	-1.395, 0.590	-0.127	0.498	-0.255	-1.106, 0.853
	Physical activity	-3.677	0.873	-4.214***	-5.394, -1.959	-2.910	0.844	-3.448**	-4.571, -1.249	-2.670	0.827	-3.23**	-4.297, -1.043
	Social contact	2.362	0.948	2.492**	0.497, 4.227	2.357	0.904	2.609**	0.579, 4.136	2.715	0.887	3.059**	0.968, 4.461
	Chronic pain	4.766	1.207	3.948***	2.390, 7.143	3.848	1.164	3.307**	1.557, 6.139	3.689	1.137	3.243**	1.450, 5.927
Self-esteem						0.769	0.142	5.425***	0.490, 1.048	0.681	0.140	4.854***	0.405, 0.957
Death anxiety										-0.170	0.044	-3.842***	-0.258, -0.083
F		7.041				10.152				11.056			
Adjusted *R²*		0.142				0.219				0.256			

*Note*: **represents *P* < 0.01,***represents *P* < 0.000

### Test of the mediating effect of death anxiety on self-esteem and quality of life

**Fig. 3:**
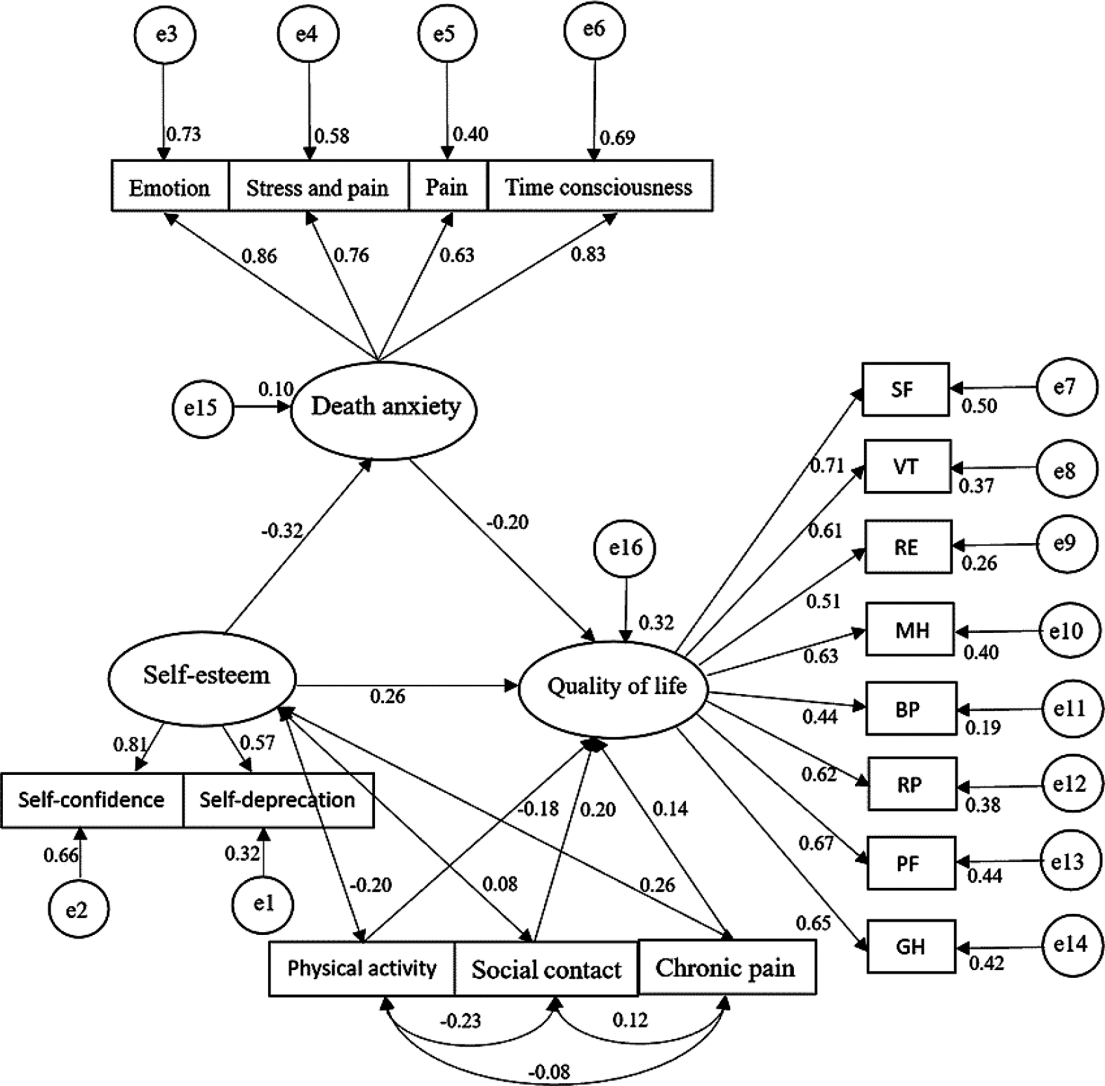
The structural equation model

**Fig. 4:**
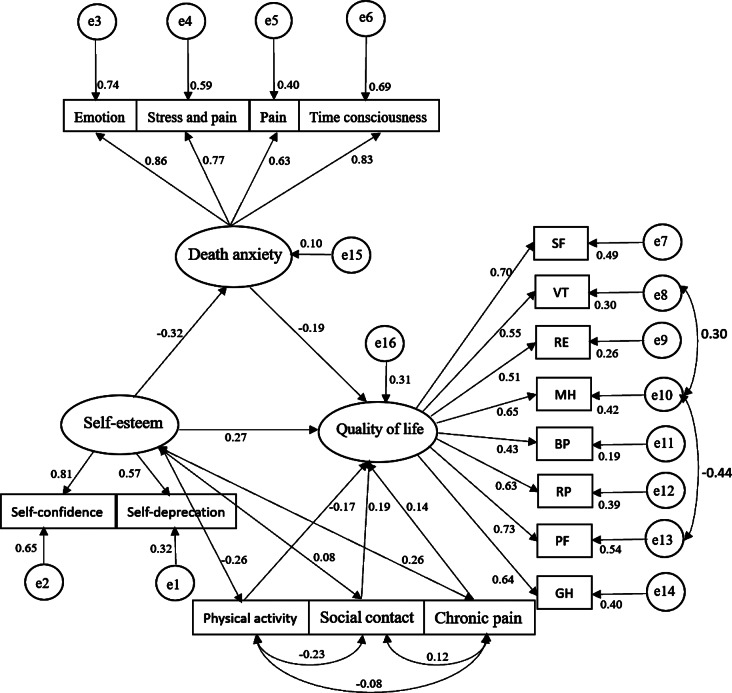
The modified structural equation model

A structural equation model with physical activity, social activity, and chronic pain as control variables; self-esteem as the independent variable; death anxiety as the mediator; and quality of life as the dependent variable was constructed using AMOS 23.0, and the structural model was estimated and tested using the method of great likelihood (Fig. 3). Two MI corrections were applied to the model based on the correction indicator, and the results showed (Fig. 4) that the model fitness was acceptable: CMIN/DF = 2.659, GFI = 0.913, TLI = 0.900, CFI = 0.903, IFI = 0.905, RMSEA = 0.069, SRMR = 0.065, PGFI = 0.638, PNFI = 0.666. By repeating the sampling 5000 times, a bias-corrected nonparametric percentile Bootstrap approach with 95% confidence intervals was used to validate the intermediate effect of death anxiety. The results showed (Table 4) that the direct effect (β = 0.266, SE = 0.089, 95% CI= [0.090,0.441]) accounted for 81.60% of the total effect, and the indirect effect (β = 0.061, SE = 0.026, 95% CI= [0.020,0.124]) accounted for 18.40% of the total effect. According to Bias-corrected upper and lower intervals that do not contain 0 and Z > 1.96 or Z = 1. 96, which is the criterion for proving that the indirect effect is valid. Thus, death anxiety partially mediates the relationship between self-esteem and quality of life in middle-aged and elderly patients with chronic diseases. The results of structural model assessment and hypothesis testing are shown in Table 5. All hypotheses are statistically significant through structural model assessment with t-values greater than 1.96.

**Table 4:** A test of mediating effects between self-esteem and quality of life

Path	Estimate	S.E.	Bias-corrected 95%CI		Effect Ratio (%)
			Lower	Upper	
Indirect Effect					
X→M→Y	0.061	0.026	0.020	0.124	18.40
Direct Effect					
X→Y	0.266	0.089	0.090	0.441	81.60
Total Effect	0.326	0.082	0.165	0.486	100

*Note*: X is Self-esteem; M is Death anxiety; Y is Quality of life

**Table 5:** Results of structural model assessment and hypothesis test

	Causality relationship	b	β	T	Results
H1a	Self-esteem→ Quality of life	1.578**	0.266	3.180	Accepted
H1b	Self-esteem→ Death anxiety	-1.534***	-0.318	-3.850	Accepted
H1c	Death anxiety→ Quality of life	-0.232**	-0.189	-2.840	Accepted
H2	Self-esteem-Death anxiety→ Quality of life	0.357**	0.061	2.346	Accepted

*Note*: **represents *P* < 0.01, ***represents *P* < 0.001.β: Standardized coefficient, b: unstandardized coefficient

## Discussion

This study analyzed the mediating effect of death anxiety on the correlation between self-esteem and quality of life in middle-aged and older patients with chronic conditions. The results of the study demonstrated a significant relationship between self-esteem, death anxiety, and quality of life, with death anxiety serving as a partial explanation for the relationship between self-esteem and quality of life.

The quality of life of middle-aged and elderly patients with chronic diseases is lower than that of the general urban population in China [36]. According to research, there were statistically significant disparities in the quality of life for middle-aged and senior chronic disease patients in terms of physical activity, social interactions, and chronic pain. Middle-aged and older patients with chronic conditions reported persistent or sporadic physical discomfort, which decreased their willingness to exercise. Exercise improves mental health, lowers the risk of falling, and lowers death rates in general [37, 38]. Regular exercise improved everyday functioning, allowed for the proper flexibility of various body parts, and reduced disease-related physical discomfort [37]. Therefore, physical activity is essential for improving middle-aged and elderly people’s quality of life. Reduced socialization may lead to immune system and neuroendocrine disorders that can negatively affect the body [39]. In addition, with the development of communication technologies, people are getting more emotional support and health knowledge from the Internet. However, social contacts online may differ significantly between older and middle-aged patients [40]. This difference limited their ability to learn health-related knowledge and their access to health information. Patients experience increased stress as a result of various health issues, which negatively impacts their physical and emotional well-being and lowers their quality of life. The continual expenditures of managing chronic pain may put financial strain on patients, in addition to reducing physical comfort and restricting daily activities. These issues lead to patients feeling guilty and distressed [41], which is bad for their mental health and lowers their quality of life overall.

This study showed that self-esteem levels were closely related to the quality of life of middle-aged and elderly patients with chronic diseases. Patients with low self-esteem levels typically had a lower quality of life, which was consistent with previous research findings [10]. Chronic disease traits, including recurring episodes and numerous complications, might make patients believe their illnesses are uncontrollable and lower their self-esteem [42]. In contrast to patients with high self-esteem, people with low self-esteem have a tendency to secrete excessive amounts of the stress hormone cortisol when under stress. The hypothalamic-pituitary-adrenal (HPA) axis can be disrupted by excessive cortisol hormone. That can then alter the regulation of other physiological systems (including the immune system) and have an impact on the body’s metabolic processes, causing further harm to the patient’s health conditions [43, 44]. Reduced self-esteem causes psychological stress to rise in middle-aged and older chronic disease patients, who then turn to unhealthy coping mechanisms [45, 46]. Patients struggle to adjust to social duties and achieve self-management requirements as a result, and this eventually lowers their quality of life. On the other hand, those with high levels of self-esteem often lead better lives [9]. More positive emotions are frequently present in patients who have high levels of self-esteem, and this can lessen the harmful effects of negative emotions on self-perception, emotions, and behavior [6, 45]. Additionally, having high levels of self-esteem can strengthen bonds with family and friends [47], which increases the amount of social support that patients receive. Those boost patients’ confidence in their ability to fight the illness, handle psychological distress logically, and adopt proactive treatment modalities, thus boosting their quality of life.

Middle-aged and elderly patients’ levels of self-esteem have an impact on their quality of life both directly and indirectly through their anxiety about dying. Terror Management Theory (TMT) claims that fear of death is a natural human reaction and that the knowledge that people will inevitably die generates death anxiety [48]. Low levels of death anxiety can trigger the body’s proximal defenses, meaning they protect people from the threat of death anxiety by distracting them from it and preventing them from thinking about it while preserving a steady quality of life. On the contrary, high degrees of death anxiety might result in phobias, eating disorders, and psychiatric problems [49]. Middle-aged and elderly patients with chronic diseases will distrust medical professionals’ treatment recommendations when asking about health issues, increasing psychological pressure, lowering confidence in life, and further developing poor coping mechanisms, all of which will lower quality of life [49–51]. Self-esteem acts as a buffer for death anxiety, and when people are reminded of death, they respond more positively with a stronger need for self-esteem [52, 53]. This can be explained by the dual-processing model of death anxiety: individuals with high self-esteem have the capacity to quickly assume social roles and seek to minimize the discrepancies between their true selves and cultural norms in order to satisfy their ego. They also maintain their composure in the face of death anxiety and psychologically sublimate their identities to shield themselves from the psychological and physical risks connected with death anxiety, maintaining their quality of life [21, 54]. Patients with low self-esteem, on the other hand, find it challenging to restrain their emotions while they are experiencing death anxiety. Instead, they magnify unpleasant emotions and practice bad habits like avoiding social situations and themselves [13, 53]. Their quality of life may be severely impacted by those. In addition, Middle-old-aged patients who have low levels of self-esteem may experience high levels of death anxiety, which may worsen their sense of self-awareness and undermine their belief in their own worth. This vicious cycle between low self-esteem and high death anxiety speeds up the decline in their quality of life.

Based on the study, it is crucial that clinical healthcare providers pay close attention to middle-aged and older patients with chronic diseases’ self-esteem and death anxiety in future treatments and care. It is recommended that patients strengthen their connections with family and friends, establish healthy relationships, and gain a sense of meaning and value in life [55]. Furthermore, social psychology education and self-management education programs or lectures, as well as cognitive-behavioral therapy, should be implemented to enhance patients self-worth and improve their self-esteem. Death culture and death education courses should be offered in hospitals and communities to help patients better understand the meaning of death, and cognitive-behavioral therapy can be used to alleviate death anxiety [56]. In addition, in future clinical work, it is suggested that medical workers strengthen education for middle-aged and elderly chronic patients. Patients should be encouraged and guided to engage in regular physical exercises such as Tai Chi and Ba Duan Jin, depending on their health conditions. Patients should also be encouraged to participate in social and recreational activities to provide them with social support. Social-psychological interventions, such as mindfulness therapy [56], should be provided for chronic pain patients to improve their quality of life. Through these measures, it is hopeful that we can improve the quality of life of middle-aged and elderly patients with chronic diseases.

## Limitation

This research has several limitations. First off, some covariates were not sufficiently accounted for, despite factors like age, education, and marital status having been taken into account. To lessen interference with the findings, tighter control over other variables should be used in future studies. Second, this study could only represent one region because it used sample data from Zhejiang Province. However, if we take into account gathering data from various places and boosting the sample size, this issue might be resolved.

## Conclusion

Our data provide evidence that self-esteem and death anxiety are factors that influence the quality of life of middle-aged and elderly patients with chronic diseases. It also verifies the partial mediating role of death anxiety between self-esteem and quality of life. Therefore, interventions that enhance self-esteem and reduce death anxiety should be implemented to improve the quality of life of patients. Furthermore, this study also finds that physical activity, social activities, and chronic pain have an impact on quality of life. Hence, when formulating intervention strategies, it is necessary to design personalized interventions based on individual characteristics in order to effectively promote the psychological and physical health of patients and improve the quality of life of middle-aged and elderly patients with chronic diseases.

## Data Availability

The datasets used and analyzed during the current study are available from the corresponding author on reasonable request.

## Abbreviations

CMIN: cardinality
DF: Degrees of Freedom
GFI: Goodness of Fit Index
TLI: Taker-Lewis Index
CFI: Comparative fit index
IFI: Invaluable Adaptation Index
RMSEA: Absolute Fitness index
SRMR: Standardized Root Mean Square Residual
PNFI: Parsimonious Normative Fit Index; PGFI:Parsimonious Goodness of Fit Index
